# Cytotoxic reactions of CC531s towards liver sinusoidal endothelial cells: a microscopical study

**DOI:** 10.1186/1476-5926-2-S1-S49

**Published:** 2004-01-14

**Authors:** Katrien Vekemans, Maarten Timmers, David Vermijlen, Ronald De Zanger, Eddie Wisse, Filip Braet

**Affiliations:** 1 Laboratory for Cell Biology and Histology, Free University of Brussels (VUB), Laarbeeklaan 103, 1090 Brussels, Belgium; 2 Present address: Department for Molecular Biomedical Research, Molecular Cell Biology Unit, Ghent University (UGhent), Technologiepark 927, 9052 Zwijnaarde, Belgium

## Introduction

Colorectal cancer cells can induce apoptosis in cells of various tissues [1]. Apoptosis can be induced by a number of factors such as Fas inducing apoptosis through the Fas/FasL pathway; other factors involve the TRAIL pathway and TNF. It is known that metastasizing colon cancer cells express more FasL then primary carcinoma cells [2]. We investigated whether a rat colon carcinoma cell line CC531s could induce apoptosis in liver sinusoidal endothelial cells (LSECs). LSECs and CC531s were co-cultured for 18 hrs and cells were visualized by SEM and TEM. Apoptosis was visualized by markers such as Hoechst and Propidium iodide. Furthermore, cells were recorded by time lapse video microscopy with and without an antagonistic antibody for FasL.

## Methods

### Animals, antibodies & reagents

Male Wistar rats (8–12 weeks old) were purchased at the Center of Laboratory Animals (Leuven, Belgium). Animals had free access to food and water. 3,3'-dioctadecyloxacarbocyanine perchlorate (DiO) (catalogue no. D-275), Hoechst 33342 (HO 33342) (Catalogue no. H-3570) and Propidium iodide (PI) (catalogue no. P-3566) were purchased at Molecular Probes (Leiden, The Netherlands). Anti-FasL blocking antibody, MFL-4, was obtained from BD Pharmingen (Erembodegem, Belgium, catalogue no. 555021).

### Co-culture of CC531s cells and LSECs

LSECs were isolated [3], purified and seeded to confluence on collagen-S coated wells and RPMI- 1640 supplemented with 10% heat inactivated fetal calf serum (FCS), 2 mM L-glutamine, 100 U/ml penicillin and 100 micrograms/ml streptomycin. The cells were kept in culture for 4 hrs. Subsequently, cells were washed with prewarmed RPMI. CC531s cells were seeded on top of LSECs at a ratio of 1:10. In order to visualize apoptosis, cells were stained with Hoechst 33342 and propidium iodide [4]. In order to discriminate cells, CC531s were stained with DiO [5]. For TEM and SEM, cells were prepared [6] and viewed with F. E. I. Tecnai 10 and Philips SEM 505. For time lapse recordings, cells were recorded up to 1 hr. Cells were cultured in medium only and/or with an antagonistic antibody against FasL (20 micrograms/ml).

## Results and Discussion

**Figure 1:**
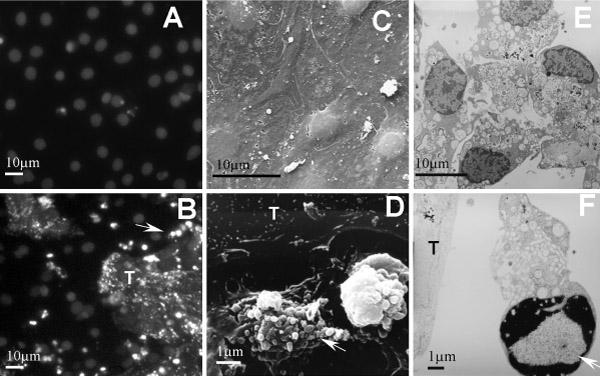
LSECs in control conditions (A, C, E) and co-culture of CC531s cells (T) and LSECs (B, D, F). A, B: Hoechst and propidium iodide staining recorded with a fluorescence microscope, Control LSECs (A) have almost no fragmented nuclei while LSECs (B) neighboring CC531s also reveal fragmented nuclei (arrow). CC531s are stained with DiO to differentiate from the LSECs. C, D: SEM recordings, in control cells (C) fenestrae can be observed and cytoplasm of the cells is well spread while CC531s neighboring the LSECs induce apoptosis in LSECs (D), as shown by blebbing and retraction of the cytoplasm (arrow). E, F: TEM recordings, control cells (E) show sponge like cytoplasm while LSECs (F) with CC531s cells show fragmented nuclei (arrow).

After 18 hrs of co-culture of LSECs and CC531s, LSECs reveal typical features of apoptosis (Fig. 1). After Hoechst and propidium iodide staining LSECs neighboring the CC531s cells reveal fragmented nuclei, in contrast to LSECs cultures where the nuclei display a normal morphology. With electron microscopy the same observation could be made, the surface of the cells reveals blebbing and with the TEM fragmented nuclei could be observed. Both in comparison with the control condition where LSECs reveal fenestrae and sponge-like appearance of the cytoplasm. In the literature, this type of apoptosis was also observed when human umbilical vein endothelial cells HUVEC and a mammary carcinoma cell line [7].

**Figure 2:**
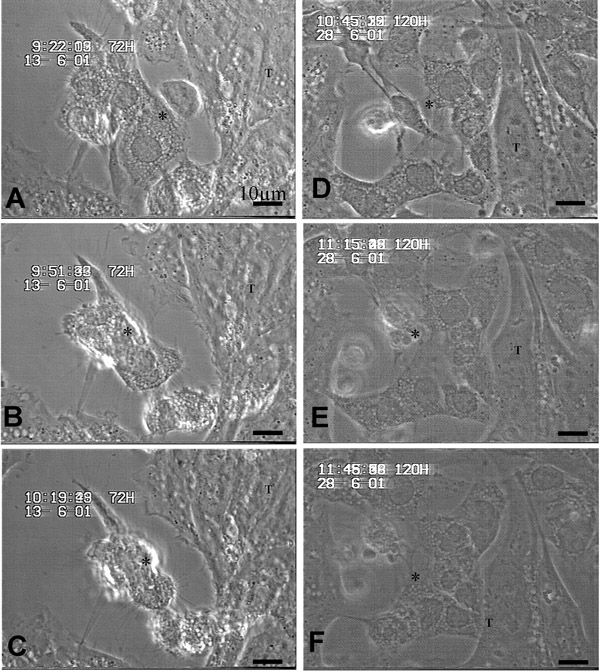
LSECs (*) and CC531s (T) co-cultures were performed with (D-F) and without aFasL (A-C) After 18 hrs incubation, recordings were made for 1 hour at time points 0 (A, D), 30 (B, E) and 60 (C, F) minutes are shown here. In conditions without aFasL, cells are rounding up and reveal signs of apoptosis after contact with CC531s. In conditions with aFasL, LSECs remain unchanged.

Time-lapse recordings (Fig. 2) provided comparable observations in the non-treated cells. At time point 0, LSECs reveal spread cytoplasm. After 30 minutes, cells start to shrink and after 1 hr cells show signs of apoptosis. In contrast, treated cells remain to have a spread cytoplasm and do not proceed to apoptosis. In accordance, colon carcinoma cell lines can also induce apoptosis in Fas bearing hepatocytes [8].

In conclusion: CC531s cells can induce apoptosis in LSECs and and antagonistic antibody against FasL abrogates this apoptosis.
